# Retraction Note: Investigation of phase transformations and corrosion resistance in Co/CoCo_2_O_4_ nanowires and their potential use as a basis for lithium-ion batteries

**DOI:** 10.1038/s41598-026-51514-x

**Published:** 2026-05-11

**Authors:** M. V. Zdorovets, A. L. Kozlovskiy

**Affiliations:** 1 The Institute of Nuclear Physics of the Republic of Kazakhstan, Almaty, Kazakhstan; 2 L.N. Gumilyov Eurasian National University, Nur-Sultan, Kazakhstan; 3 Ural Federal University, Yekaterinburg, Russia; 4 Kazakh-Russian International University, Aktobe, Kazakhstan

Retraction of: *Scientific Reports* 10.1038/s41598-019-53368-y, published online 12 November 2019

The Editors have retracted this Article.

After publication, concerns were raised regarding the veracity of data presented in this work. Specifically, the EDS spectrum shown in Figure 1b appears to have repetitive sections of background noise. The Authors provided some raw data on request from the Editors, but this was insufficient to address the concerns raised. The Editors therefore no longer have confidence in the research presented in this Article.

The Authors did not respond to the correspondence from the Editors about this retraction.

